# Dietary Prebiotics and Bioactive Milk Fractions Improve NREM Sleep, Enhance REM Sleep Rebound and Attenuate the Stress-Induced Decrease in Diurnal Temperature and Gut Microbial Alpha Diversity

**DOI:** 10.3389/fnbeh.2016.00240

**Published:** 2017-01-10

**Authors:** Robert S. Thompson, Rachel Roller, Agnieszka Mika, Benjamin N. Greenwood, Rob Knight, Maciej Chichlowski, Brian M. Berg, Monika Fleshner

**Affiliations:** ^1^Stress Physiology Laboratory, Department of Integrative Physiology, University of Colorado at BoulderBoulder, CO, USA; ^2^The Center for NeuroscienceUniversity of Colorado at Boulder, Boulder, CO, USA; ^3^Department of Psychology, University of Colorado at DenverDenver, CO, USA; ^4^Department of Pediatrics, University of California School of MedicineSan Diego, CA, USA; ^5^Pediatric Nutrition Institute, Mead Johnson NutritionEvansville, IN, USA

**Keywords:** sleep, stress, prebiotics, lactoferrin, MFGM, nutrition, temperature

## Abstract

Severe, repeated or chronic stress produces negative health outcomes including disruptions of the sleep/wake cycle and gut microbial dysbiosis. Diets rich in prebiotics and glycoproteins impact the gut microbiota and may increase gut microbial species that reduce the impact of stress. This experiment tested the hypothesis that consumption of dietary prebiotics, lactoferrin (Lf) and milk fat globule membrane (MFGM) will reduce the negative physiological impacts of stress. Male F344 rats, postnatal day (PND) 24, received a diet with prebiotics, Lf and MFGM (test) or a calorically matched control diet. Fecal samples were collected on PND 35/70/91 for 16S rRNA sequencing to examine microbial composition and, in a subset of rats; *Lactobacillus rhamnosus* was measured using selective culture. On PND 59, biotelemetry devices were implanted to record sleep/wake electroencephalographic (EEG). Rats were exposed to an acute stressor (100, 1.5 mA, tail shocks) on PND 87 and recordings continued until PND 94. Test diet, compared to control diet, increased fecal *Lactobacillus rhamnosus* colony forming units (CFU), facilitated non-rapid eye movement (NREM) sleep consolidation (PND 71/72) and enhanced rapid eye movement (REM) sleep rebound after stressor exposure (PND 87). Rats fed control diet had stress-induced reductions in alpha diversity and diurnal amplitude of temperature, which were attenuated by the test diet (PND 91). Stepwise multiple regression analysis revealed a significant linear relationship between early-life *Deferribacteres* (PND 35) and longer NREM sleep episodes (PND 71/72). A diet containing prebiotics, Lf and MFGM enhanced sleep quality, which was related to changes in gut bacteria and modulated the impact of stress on sleep, diurnal rhythms and the gut microbiota.

## Introduction

Exposure to stress negatively affects sleep and the sleep/wake cycle. For example, experiencing work-related stressors (Akerstedt et al., 2002), having low social support (Mellman and Hipolito, 2006), or exposure to trauma/combat (Mellman et al., 1995, 2007; Rothbaum and Foa, 2002; Capaldi et al., 2011) can all disrupt sleep and the sleep/wake cycle. Preclinical studies testing a variety of animal models of stress in early life or adulthood also report similar outcomes on sleep and the sleep/wake cycle (Madan et al., 2008; Pawlyk et al., 2008; McClung, 2011; Yang et al., 2011; Greenwood et al., 2014; Thompson et al., 2016).

Stressor exposure, including diurnal rhythm disruption (Thaiss et al., 2014), can produce gut microbial imbalance or dysbiosis (Louis and O’Byrne, 2010). Commensal gut bacteria play an important role in many aspects of host health and physiology (Galland, 2014; Mayer et al., 2014; Putignani et al., 2014; Salzman, 2014; Vitetta et al., 2014); and early-life gut microbial dysbiosis is associated with increased risk of allergic disease, colitis and gut inflammation (Bisgaard et al., 2011; Voigt et al., 2014).

There is evidence that stressor exposure produces dysbiosis by impacting specific gut bacteria and producing community structure changes, including reductions in alpha diversity. Alpha diversity is a measure of bacterial community richness (the number of unique bacterial taxa), and evenness (how evenly the abundance of the different taxa are distributed). Maternal separation of infant monkeys, for example, produces a selective reduction in gut *Lactabacilli* (Bailey and Coe, 1999); and rats exposed to tail shock stress have rapid reductions of *Provetella* measured in both fecal and cecum samples (Maslanik et al., 2012a). Whereas, mice exposed to chronic social disruption (Bailey et al., 2010, 2011), circadian disruption plus alcohol consumption (Voigt et al., 2016) or 8 weeks of circadian disorganization plus a high fat diet (Voigt et al., 2014) have clear reductions in alpha diversity. Thus, stressor exposure induces dysbiosis by impacting specific bacteria and by reducing measures of alpha diversity. Taken together, stressor exposure can negatively affect sleep, the sleep/wake cycle and the gut microbiota community structure.

One approach used to alleviate the negative effects of stressor exposure on the gut microbiota community structure is through prophylactic administration of probiotics. Certain probiotics, such as *Lactobacilli* spp. and *Bifidobacteria* spp., can modulate brain function and stress responses (Gibson and Roberfroid, 1995; Forsythe and Kunze, 2013; Saulnier et al., 2013; Galland, 2014; Burokas et al., 2015; Dash et al., 2015; Neuman et al., 2015; Zhou and Foster, 2015; Monteagudo-Mera et al., 2016). In humans, for example, consumption of *Lactobacillus* spp. and *Bifidobacteria* spp. significantly reduced self-reported cognitive reactivity to sad mood in depressed individuals, thus alleviating stress-induced symptoms (Steenbergen et al., 2015). In addition, *Lactobacillus reuteri* administered to infants reduced colic and colic-associated maternal depression (Mi et al., 2015).

Similarly, in rodent models *Lactobacillus rhamnosus* reduced anxiety and depressive-like behavior and exaggerated HPA axis activation (Bravo et al., 2011). *Lactobacillus farciminis* attenuated stress-evoked HPA responses (Ait-Belgnaoui et al., 2012) and *Lactobacillus plantarum* attenuated early-life stress-induced depressive behavior (Liu et al., 2016). Finally, *Bifidobacterium longum* was also reported to reduce anxiety-like behavior in the innately anxious BALBc mice (Savignac et al., 2014). Both the clinical and preclinical literatures suggest that certain types of probiotics, like *Lactobacilli* spp. and *Bifidobacteria* spp., can modulate brain function and stress responses.

Another more novel approach that may yield long-term benefits to the gut microbiota community structure is to administer prebiotics in the diet, specifically starting early in life. Prebiotics are non-digestible fibers that provide nutrients to facilitate expansion of select types of resident bacteria, including *Lactobacilli* spp. and* Bifidobacteria* spp. Infants fed the prebiotic, galactooligosaccharides (GOS), for example, had increased fecal *Bifidobacteria*, and adults fed GOS had attenuated stress-induced neuroendocrine responses (Schmidt et al., 2015) and gastrointestinal distress (Witaicenis et al., 2010; Caporaso et al., 2011). In addition, mice fed the prebiotics 3′Sialyllactose and 6′Sialyllactose had reduced anxiety-like behavior tested in the open-field (Tarr et al., 2015) and GOS reduced anxiety-like behavior produced after an immune challenge stressor (Savignac et al., 2016). Thus prebiotic nutrient administration can influence the gut microbiota and some features of the systemic stress response.

Additional dietary components lactoferrin (Lf) and milk fat globule membrane (MFGM) can also impact the gut microbiota. A recent study reported an association between the concentration of human milk Lf, which has antimicrobial properties, and the abundance of *Bifidobacteria* and *Lactobacilli* in breast-fed infants (Mastromarino et al., 2014). Thus, Lf may also help promote the growth of beneficial bacteria in the host, specifically in early-life. MFGM, another bioactive component of human milk, also provides a variety of important nutritional, antimicrobial and cognitive benefits (Dewettinck et al., 2008; Chatterton et al., 2013; Timby et al., 2014). Together these studies suggest that dietary prebiotics, Lf and MFGM can impact the gut microbiota and potentially promote stress resistance (Sarao and Arora, 2015).

While it is clear that exposure to stress can affect the sleep/wake cycle and can negatively impact the commensal gut bacteria, it remains unknown whether a diet rich in prebiotics started early in life can promote a stress protective phenotype that includes protection of the sleep/wake cycle. Therefore, the current study tested the hypothesis that consumption of a test diet GOS, polydextrose (PDX), Lf and MFGM compared to calorically matched control diet would reduce the impact of stressor exposure on the sleep/wake cycle and the gut microbiota community structure.

## Materials and Methods

### Animals

Adult male F344 rats (*n* = 52, Harlan Laboratories) weighing 40–50 g at arrival, post natal day (PND) 24; were housed with controlled temperature (22°C) and humidity. We selected F344 rat strain because they are an inbred strain known to respond to stressors in a consistent fashion and allow us to minimize group size. The goal of this study was to explore the potential protective effects of a prebiotic diet on stress-induced disruptions in sleep and physiology. Using the F344 rat strain allowed us to maximize the impact of stressor exposure, minimize the group sizes and reduce variability in physiological and microbial dependent measures. The animals were maintained on a 12:12 h light/dark cycle (lights on 04:00–16:00 h). All rats were housed in Nalgene Plexiglas cages (45 cm × 25.2 cm × 14.7 cm) and were allowed to acclimate to the housing conditions for 1 week. Rats had *ad libitum* access to food and water and were weighed once per week. All experimental procedures were performed during the inactive cycle and animals were handled during the 1 week acclimation period. Animal discomfort was minimized during all procedures. Given that rats arrived at such a young age (PND 24) they were double housed prior to surgical implantation of the biotelemetry device to minimize discomfort. After surgery rats were single housed as necessary to record from telemetry devices. Rats were started on either control diet or test diet immediately upon arrival at PND 24. We chose to start them at such an early age because it has been demonstrated that the gut bacteria can be more easily shaped by exogenous factors such as exercise and a prebiotic diet very early in life, which leads to what is thought to be a stress-protective gut bacteria pheontype (Mika et al., 2015b; Mika and Fleshner, 2016). Additionally, starting the prebiotic diet as early as PND 24 has been shown to alleviate the negative consequences of stress in adulthood (Mika et al., 2016). All of these effects were demonstrated in the F344 rat strain. Experimental protocols for these studies were approved by the University of Colorado Animal Care and Use Committee.

### Control and Test Diets

All experimenters were blind to type of diet administered and the diets were coded as diets 66 and diet 89. The diets were formulated by Mead Johnson Nutrition (MJN, Evansville IN, USA) based on AIN-93G specifications, were isocaloric with similar carbohydrate, protein, fat, vitamin and mineral levels. The test diet differed from control diet by the inclusion of GOS 21.23 g/Kg; PDX 6.58 g/Kg; Lf 1.86 g/Kg and Whey protein concentrate MFGM-10 15.9 g/Kg. Upon completion of the experiments and subsequent data analysis, experimenters’ were informed of the contents of diet 66 and diet 89. Diet 89 (control diet) consisted of: Casein 200.00 g/Kg; L-Cysine 3.0 g/Kg; Corn Starch 398.786 g/Kg; Maltodextrin 132.00 g/Kg; Sucrose 100.00 g/Kg; Lactose monohydrate 8.05 g/Kg; Soybean Oil 62.7 g/Kg; Cellulose 50.0 g/Kg; Mineral Mix (without Ca and P) 13.4 g/Kg; Calcium Carbonate 7.25 g/Kg; Calcium Phosphate dibasic 7.0 g/Kg; Vitamin Mix AIN-93-VX 10.0 g/Kg; Choline Bitartate 2.5 g/Kg TBHQ (antioxidant) 0.014 g/Kg; DHA/ARA Oil 5.3 g/Kg). Diet 66 (test diet) consisted of: Casein 180.00 g/Kg; L-Cysine 3.0 g/Kg; Corn Starch 383.816 g/Kg; Maltodextrin 132.00 g/Kg; Sucrose 100.00 g/Kg; Soybean Oil 60.2 g/Kg; Cellulose 50.0 g/Kg; Mineral Mix (without Ca and P) 13.4 g/Kg; Calcium Carbonate 6.9 g/Kg; Calcium Phosphate dibasic 7.3 g/Kg; Vitamin Mix AIN-93-VX 10.0 g/Kg; Choline Bitartate 2.5 g/Kg TBHQ (antioxidant) 0.014 g/Kg; DHA/ARA Oil 5.3 g/Kg; Galactooliosaccharide (fiber) 21.23 g/Kg; PDX (fiber) 6.58 g/Kg; Lf 1.86 g/Kg; Whey protein concentrate MFGM-10 15.9 g/Kg).

### Experimental Procedures: Fecal Culture Study

To verify that the test diet would increase probiotic species consistent with previous reports, a subset of rats (PND 24) were placed on either the control diet (*n* = 11) or the test diet (*n* = 9). After 4 weeks (PND 52), fecal samples were collected and selective bacterial culture was performed as described below.

#### Fecal Sample Collection

Each rat was placed into a sterile cage until defecation and samples were collected immediately thereafter (Restrepo and Armario, 1987). Most rats defecated upon placement into the cage within 5–10 min and were subsequently returned to their home cage. Several instances did occur where it took 20 min for the rats to defecate in the sterile cage although these were the exceptions, not the normal. Sterile forceps (100% ethanol) were used to obtain each sample, which were then placed in a 1.5 mL sterile screw cap tubes (USA Scientific, Ocala, FL, USA) and on ice. Samples were then transferred and stored at −80°C for later analyses. Rats were returned to their home cage immediately after sample collection. All sample collections occurred at approximately 0900.

#### Selective Bacterial Culture

Lactobacillus-specific culture media (modified-rhamnose-2,3,5-triphenyltetrazolium (TTC) chloride-LBS-vancomycin agar; M-RTLV-agar) was adapted from Sakai et al. (2010), which facilitates the selective growth and visualization of vancomycin resistant *L. rhamnosus* colony forming units (CFU). M-RTLV agar was prepared by combining L-rhamnose (0.4 g/mL), TTC (30.0 mg/mL), vancomycin (10.0 mg/mL) and metronidazole (10.0 mg/mL) with nutrient agar.

Fecal samples were homogenized (0.2 g of each sample in 2.0 mL phosphate buffered saline) and subsequently diluted in phosphate buffered saline (1:5000) and plated on M-RTLV-agar. Plated samples were incubated at 37°C; *Lactobacillus rhamnosus* anerobic bacteria were incubated in anerobic conditions created by a BD GasPak EZ Anaerobe Container System Sachets and placing the indicator inside a BD GasPak EZ Large Incubation Container (33.35 cm × 16.51 cm × 17.145 cm). After 48 h of incubation, the CFUs were counted using a cell counter (Scienceware Electronic Colony Counter) and dilution corrected averages were then calculated and analyzed. Based on color and shape, *Lactobacillus rhamnosus* colonies were isolated, sequenced by GeneWiz and verified in the SILVA data base.

### Experimental Procedures: Sleep and Gut Microbial Composition Study (Figure 1)

**Figure 1:**
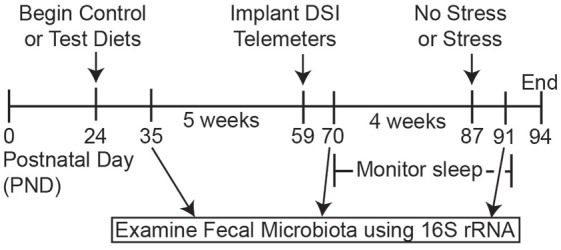
**Timeline.** Schematic depicting the experimental timeline. Rats arrived on postnatal day (PND) 24 and were immediately placed on either the control or test diets. Fecal samples for 16S rRNA analysis were collected at PND 35, PND 70 and PND 91. Biotelemetry devices were implanted at PND 59 and 10 days later sleep, body temperature and locomotor activity (LA) measures were continuously recorded until the end of the experiment. On PND 87 rats were exposed to inescapable stress and were placed back in home cages to monitor how the test diet modulated sleep recovery following stress exposure. Finally, body weights, food weights and fecal samples were taken on PND 91 to assess the impact of stress exposure on gross physiology measures and the gut microbiota structure.

The experimental design is presented in Figure 1. The group sizes used for sleep analysis were as follows: control diet (*n* = 15) and test diet (*n* = 14) at time points PND 35 and PND 70; After stress the group sizes were control diet, no stress (*n* = 7), control diet, stress (*n* = 8), test diet, no stress (*n* = 7), and test diet, stress (*n* = 7) for PND 87. Rats continued on the diets throughout the study from PND 24—PND 94 (Figure 1). Weekly food consumption, body weights and fecal pellets were obtained throughout the study. At the end of week 5 (PND 59) rats were implanted with biotelemetry devices to record locomotor activity (LA), body temperature and sleep wake, non-rapid eye movement (NREM), and rapid eye movement (REM) sleep. Recordings began 10 days after surgery (PND 69). In order to obtain accurate and undisturbed diurnal data (i.e., disruptions caused by the experimenters when weighing/changing food, weighing rats and collecting fecal pellets weekly) three separate 48-h periods during development were chosen for detailed analysis: PND 71, 72; PND 78, 79; and PND 85, 86. Stress exposure occurred on PND 87 and recording continued until the end of the study on PND 94. Fecal samples for 16S rRNA analysis were collected on PND 35 (prior to telemetry implants and stress), PND 70 (after telemetry implants but prior to stress exposure), and PND 91 (4 days after stress). Only these 3 days were chosen for 16S rRNA analysis due to the high cost of performing this analysis.

#### Biotelemetry Surgeries

The F40-EET biotelemetry transmitters (Data Sciences International, St.Paul, MN, USA) were implanted into animals as previously described (Thompson et al., 2012, 2013, 2014, 2016; Greenwood et al., 2014). Briefly, animals were fully anesthetized and unresponsive following ketamine (i.p. 75.0 mg/kg), and medetomidine (i.p. 0.5 mg/kg). Animals were shaved and prepped for surgery. A midline incision was made approximately 5.0 cm in length on the ventral abdominal wall. The F40-EET transmitter was placed on the intestines, the biopotential leads were passed through the ventral abdominal wall and then the F40-EET transmitter was sutured to the ventral abdominal wall. Once the transmitter was sutured into place, the electromyograph (EMG) leads were positioned on the back of the neck to measure skeletal muscle activity. Finally, the electroencephalographic (EEG) leads were placed as previously described. Briefly, insulated leads were passed subcutaneously to the base of the skull, where they were attached to stainless steel screws (Plastics One Inc.) and served as EEG recording electrodes. Using sterile technique, a hole was drilled to secure the screws in place at stereotaxic coordinates relative to bregma: anterior 2.0; lateral 2.5 and posterior −5.5; lateral 3.0. Once screws and leads were in place, they were embedded in dental acrylic to ensure the integrity of the recording signal. Immediately following surgery rats were given meloxicam (i.p. 1.0 mg/kg) for analgesia after which they recovered on a heating pad at 37°C until ambulatory. Animals were administered 1.0 mL of antibiotics (combi-pen-48; Penicillin G Benzathine and Penicillin G Procaine; Bimeda Inc., Le Sueur, MN, USA) subcutaneously immediately after surgery, but prior to waking from anesthesia. Once rats were self-ambulatory, they were placed back into clean home cages.

#### Stress Protocol

On PND 87, half of the rats were exposed to 100 inescapable tail shocks which is known to induce depression/anxiety-like behavior, to substantially increase corticosterone, and to disrupt diurnal physiology/sleep as previously described (Greenwood et al., 2005, 2014; Thompson et al., 2013, 2014, 2016; Speaker et al., 2014). Inescapable tail shock can produce clear impacts on the dependent measures we examined in this experiment (i.e., both sleep and the gut microbiota). Additionally, this method of stress can produce consistent impacts on our dependent measures that are easily replicated with respect to other forms of more variable stress exposure (i.e., social defeat). Briefly, on the day of exposure to tail shock, rats were taken to a separate room, placed in Plexiglas restraining tubes (23.4 cm long and 7.0 cm in diameter) and exposed to 100, 5 s, 1.5 mA inescapable tail shocks. Shocks were delivered at random with an average interval of 60-s between shocks and occurred during the inactive (light) cycle between ~08:00 h and 11:00 h. After exposure to inescapable tail shock rats were immediately returned to their home cages where continuous uninterrupted biotelemetry recording continued for 6 days. The inescapable tail shock apparatus delivers electrical current through a small portion of the tail and movement during tail shock stress is restricted, but not eliminated, by the Plexiglas tube. The degree of movement during tail shock has been previously biotelemetrically quantified (Thompson et al., 2014).

#### Data Acquisition and Analysis

The F40-EET transmitter allows *in vivo* real-time measurement of LA, core body temperature (CBT) and EEG in freely behaving animals. Biotelemetry recordings were acquired/analyzed using Dataquest ART 4.3 Gold Acquisition/Analysis Software (Data Sciences International, St. Paul, MN, USA) as previously described (Thompson et al., 2012, 2013, 2014, 2016). LA, heart rate and EEG were recorded at 500 Hz. Analysis of the sleep/wake cycles was performed using the automated Neuroscore 2.1.0 software (Data Sciences International, St. Paul, MN, USA). The trace EEG signal was subjected to fast Fourier Transformation (FFT), yielding spectra between 0.5 Hz and 30 Hz in 0.5-Hz frequency bins. The delta frequency band was defined at 0.5–4.5 Hz and the theta frequency band was defined as 6.0–9.0 Hz as previously described (Olivadoti and Opp, 2008; Thompson et al., 2016). After sleep recordings were autoscored they were corrected for accuracy by an observer blind to the experimental treatment of each animal. Each recording was autoscored/corrected in 10-s epochs and classified as NREMS, REMS or WAKE on the basis of state-dependent changes in multiple parameters, including the EEG, LA, and CBT as previously described (Thompson et al., 2016). Wakefulness was defined based on low amplitude, mixed frequency (delta ≈ theta) EEG accompanied by movement and increases in CBT during wakefulness are associated with activity. NREM sleep was identified by increased absolute EEG amplitude with integrated values for the delta frequency band greater than those for the theta frequency and an absence of LA and decreasing CBT, which declines upon entry into NREM sleep. REM sleep was characterized by low amplitude EEG with integrated values for the delta frequency band less than those for the theta frequency band. Time spent in NREM/REM/WAKE was calculated as a percentage (%) of time spent in a specific behavioral state per hour (i.e., % REM, % NREM or % WAKE). Additionally, average bout durations of NREM/REM/WAKE were calculated as an average per hour. Bout durations were defined by any change in sleep/wake state for 10 s (i.e., an NREM bout was defined based on the appearance of 10-s epoch or longer of NREM and the end of that epoch was defined as the appearance of any 10-s epoch of either REM or WAKE). Thus all three sleep/wake bout durations were defined as purely NREM sleep, REM sleep or WAKE with a temporal resolution of 10 s. Finally, for NREM/REM/WAKE the total number of episodes per hour (#) was also calculated although in this experiment there were no statistically significant differences in the total number of episodes between groups at any time analyzed. All sleep/wake data were averaged into 1-h or 12-h intervals for statistical analysis.

#### 16S rRNA Gene Sequencing and Microbial Composition Analysis

Fecal samples were collected at PND 35, 70 and 91. Samples were collected, prepared as previously described (Maslanik et al., 2012b; Mika et al., 2015b), and analyzed in the Rob Knight Lab at the University of Colorado at Boulder (prior to relocation to San Diego). 16S rRNA analysis is a general maker for visualizing the gut microbial composition (Koenig et al., 2011). Briefly, after purification and precipitation to remove polymerase chain reaction (PCR) artifacts, samples were sequenced in multiplex on an Illumina HiSeq 2000. Operational taxonomic units (OTUs) were picked using a “closed reference” approach (Navas-Molina et al., 2013) GreenGenes May 2013 version was the reference database used (McDonald et al., 2012), and all sequence processing was done with QIIME v 1.8.0 (Caporaso et al., 2010) using the UCLUST algorithm (Edgar, 2010). Taxonomy and phylogeny were taken from the GreenGenes reference collection. The current experiment generated 14,207,155 sequences, where 11,016,354 were discarded because of uncorrectable barcode errors, of which 6481 were too short to read (based on default parameters set in QIIME script “split_libraries_fastq.py”) and the remaining 3,190,801 sequences were used. The resulting OTU table was rarefied at 7400 sequences/sample to correct for uneven sequencing depth due to amplification differences between samples.

Three time points were submitted for analysis (*n* = 32 samples/time point). Several rats were eliminated from further analysis due to insufficient fecal samples. The final group sizes used for all microbiome analysis were as follows: control diet (*n* = 15) and test diet (*n* = 14) at time points PND 35 and PND 70; After stress (PND 91) the group sizes were control diet, no stress (*n* = 7), control diet, stress (*n* = 8), test diet, no stress (*n* = 5) and test diet, stress (*n* = 7).

Alpha diversity was examined using three different measures: shannon entropy, species richness (measured as the number of observed species), and phylogenetic diversity measured over the whole tree (PD_whole_tree). Shannon entropy is an indicator of an evenness of the community structure and is a non-phylogenetic measurement of bacterial abundance or richness. Phylogenetic diversity was measured as the total descending branch length of the constructed phylogenetic tree for a given sample (Faith, 1994).

Beta diversity was examined with principal coordinate analysis (PCoA) using unweighted UniFrac distances. UniFrac is an algorithm that determines differences between microbial communities between samples based upon their unique branch length on a phylogenetic tree (Lozupone and Knight, 2005).

#### Statistical Analysis

Statistical analyses were conducted using Statview (SAS Institute) and the SPSS V.21 (SPSS, Chicago, IL, USA) software packages and all data are depicted with the standard error of the mean. Prior to stressor exposure, the effect of diet on baseline measures of sleep and diurnal physiology was compared using a two variable design (control diet vs. test diet). The diurnal differences were calculated by subtracting the 12-h averaged night values from the 12-h averaged day values as previously described (Thompson et al., 2012, 2013, 2014, 2016). The effect of diet on stress responses used a 2 (control diet vs. test diet) × 2 (no stress vs. stress) design. Data were examined using repeated measures ANOVA when appropriate or a two-way ANOVA when data were averaged. Alpha diversity measures and relative abundance of microbial taxa at the phylum level were subjected to normality tests (Shapiro-Wilk), and all non-normally distributed data were rank transformed. Correlation analyses were performed using normalized data when appropriate using the arc sin square root function (Fleiss et al., 1985; Varkoohi et al., 2007); however data are depicted as relative OTU’s for ease of interpretation. Correction for multiple comparisons was conducted using the Benjamini-Hochberg step down method (Benjamini et al., 2001) implemented in the QIIME 1.8.0. Data points were determined to be outliers if they failed the Grubb’s test for ouliters (Grubbs, 1969; Thompson et al., 2014). When appropriate, *post hoc* analysis was performed using Fisher’s PLSD with alpha set to *p* < 0.05.

## Results

### Early-Life Test Diet Increased *Lactobacillus rhamnosus*

**Figure 2:**
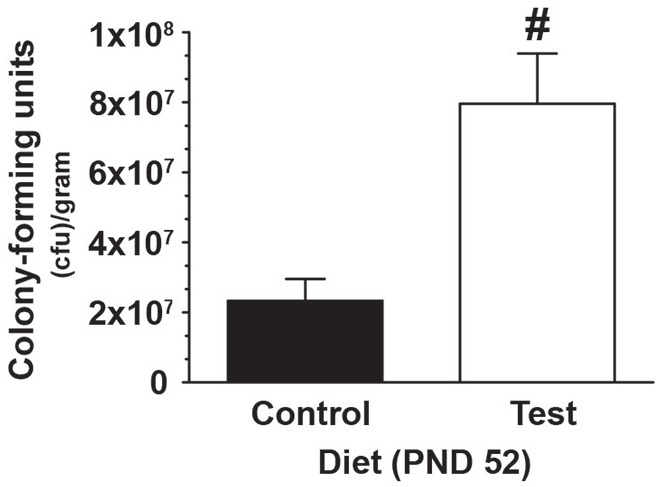
**Test diet increases *Lactobacillus rhamnosus*.** Data demonstrating, in a subset of rats, that consumption of the test diet significantly increased levels of *Lactobacillus rhamnosus* in fecal cultures at PND 52, when compared with those eating the control diet (^#^*p* < 0.05 vs. control diet).

In a subset of rats, 4 weeks of consuming a test diet compared to control diet (PND 24-PND 52) resulted in an increase in the number *Lactobacillus rhamnosus* CFU selectively cultured from fecal samples (*F*_(1,18)_ = 14.997; *p* = 0.001; main effect of diet; see Figure 2), thus demonstrating that the test diet, compared to control diet, was effective at increasing *Lactobacillus rhamnosus*.

### Test Diet Attenuated the Stress-Induced Disruption in the Diurnal Amplitude of Core Body Temperature, but did Not Affect Food Consumption or Body Weight

**Figure 3:**
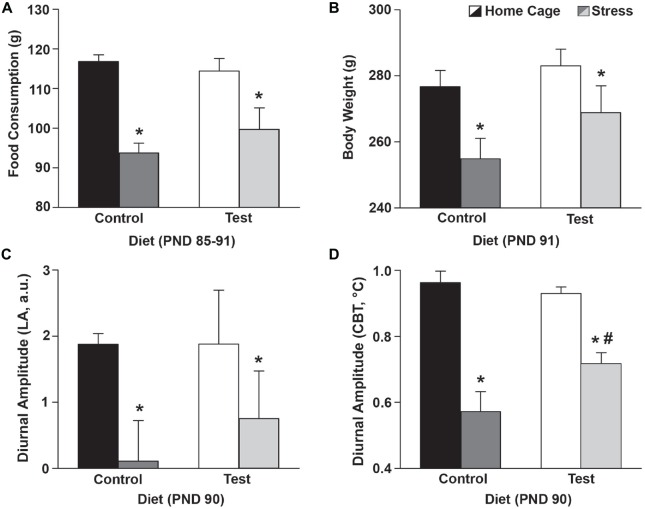
**Stress effects on food/body weight/diurnal CBT.** Food consumption, body weight and diurnal amplitude data 4 days after stress exposure. **(A)** Stress exposure reduced food consumption, but there were no differences in food consumption between the control and test diets. The food consumption depicted was a weekly average based on food consumed from PND 85–91. **(B)** Similarly, stress exposure resulted in lower body weights but there were no differences in body weight between the control and test diets. **(C)** Stress flattened the diurnal amplitude of LA of both control and test diet groups, but diet did not alter this effect. **(D)** Stress also flattened the diurnal amplitude of CBT of both control and test diet groups, however, the test diet group had an attenuated disruption in the diurnal amplitude of CBT when compared with those on the control diet. Abbreviations are as follows: a.u., arbitrary units; CBT, core body temperature; g, grams; LA, locomotor activity; PND, post natal day (*p < 0.05 vs. no stress; ^#^*p* < 0.05 vs. control diet).

Throughout development (PND 24–86) there were no significant differences in consumption of either the control diet or the test diet (*F*_(1,25)_ = 1.834; *p* = 0.188; no main effect of diet; data not shown) or between no stress/stress groups prior to the day of stress exposure (*F*_(1,25)_ = 0.000; *p* = 0.993; data not shown). Following stress exposure (PND 85–91), however; rats exposed to stress ate less (*F*_(1,25)_ = 30.538; *p* < 0.0001; main effect of stress; see Figure 3A for results of *post hoc* analysis), and diet had no effect on the stress-induced reduction of food consumption.

Four days following stress exposure (PND 91) rats exposed to stress had reduced body weights (*F*_(1,25)_ = 8.337; *p* = 0.008; see Figure 3B for results of *post hoc* analysis), however diet had no effect on the stress-induced reduction in body weight.

Stress flattened the diurnal amplitude of LA (*F*_(1,25)_ = 5.256; *p* < 0.030; main effect of stress; see Figure 3C for results of *post hoc* analysis), but diet had no effect on this stress-induced reduction. Stress also flattened the diurnal amplitude of CBT (*F*_(1,25)_ = 53.038; *p* < 0.0001; main effect of stress; see Figure 3D), however, rats eating the test diet had an attenuated disruption in the diurnal amplitude of CBT when compared to those eating a control diet at PND 90 (*F*_(1,25)_ = 4.567; *p* = 0.042; interaction between stress and diet; see Figure 3D for results of *post hoc* analysis).

### Test Diet Improved NREM Sleep Consolidation in Early-Life (PND 71, 72)

**Figure 4:**
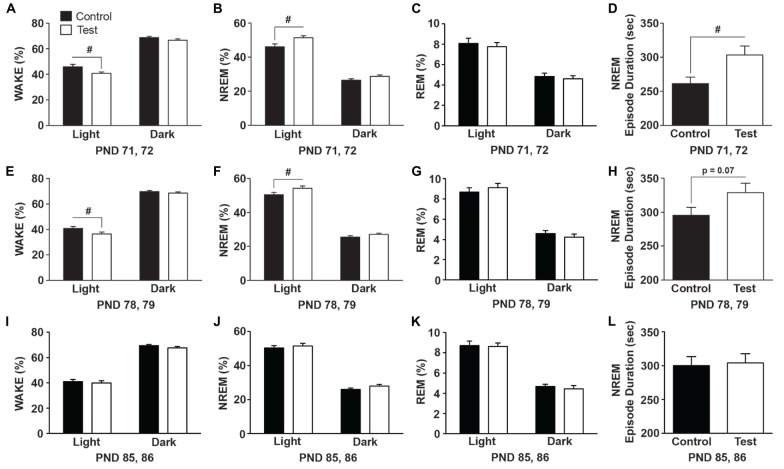
**Diet effects on sleep.** Data expressed as average amounts of the two recording days (12 h light and 12 h dark) indicating that rats consuming the test diet spent more time in NREM sleep and had increased NREM sleep consolidation in early-life. **(A)** Wake was reduced and **(B)** NREM sleep was increased during the light cycle in rats consuming test diet when compared to rats on the control diet. **(C)** There was no effect of diet on rapid eye movement (REM) sleep. **(D)** The average duration of NREM sleep episodes was significantly longer indicating greater NREM sleep consolidation in rats consuming the test diet when compared to rats on the control diet. One week later **(E)** wake was still reduced and **(F)** NREM sleep increased during the light cycle in rats consuming test diet, **(G)** there was no effect of diet on REM sleep, and **(H)** there was only a trend (*p* = 0.07) towards increased NREM sleep consolidation. The effects of diet were no longer present on **(I)** wake, **(J)** NREM sleep, **(K)** REM sleep or **(L)** NREM episode duration on PNDs 85, 86. Abbreviations are as follows: NREM, Non-rapid eye movement; PND, post natal days; sec, seconds (^#^*p* < 0.05 vs. control diet).

During early-life (PND 71, 72) rats on the test diet spent *les*s time in WAKE (*F*_(1,27)_ = 5.77; *p* = 0.023; Figure 4A) and accordingly spent *more* time in NREM sleep (*F*_(1,27)_ = 8.125; *p* = 0.008; Figure 4B), which was due to longer NREM episode durations or bouts (*F*_(1,27)_ = 6.617; *p* = 0.015; Figure 4D). There was no effect of diet on REM sleep (Figure 4C). One week later (PND 78, 79) rats on the test diet still spent *less* time in WAKE (*F*_(1,27)_ = 4.73; *p* = 0.038; Figure 4E) and *more* time in NREM sleep (*F*_(1,27)_ = 5.335; *p* = 0.028; Figure 4F); however there was only a trend towards more consolidated NREM sleep (*F*_(1,27)_ = 3.386; *p* = 0.07; Figure 4H). There was no effect of diet on REM sleep (Figure 4G). The effects of the test diet on sleep was no longer present in WAKE (Figure 4I), NREM sleep (Figure 4J) or REM sleep (Figure 4K) at PND 85, 86. Similarly, NREM sleep episode durations were equal between the control and test diets at PND 85, 86 (Figure 4L) just prior to stress exposure.

The test diet-induced changes in sleep were not due to gross differences in either LA or CBT as there was no effect of diet on either LA (*F*_(1,27)_ = 0.118; *p* = 0.734; data not shown) or body temperature (*F*_(1,27)_ = 0.863; *p* = 0.361; data not shown) when the biggest diet-induced differences in NREM sleep were observed (i.e., PND 71, 72). One week later (PND 78, 79) there was also no effect of diet on either LA (*F*_(1,27)_ = 2.183; *p* = 0.151; data not shown) or body temperature (*F*_(1,27)_ = 0.812; *p* = 0.375; data not shown).

### Test Diet Supports Beneficial REM Sleep Rebound Following Acute Stress (PND 87)

**Figure 5:**
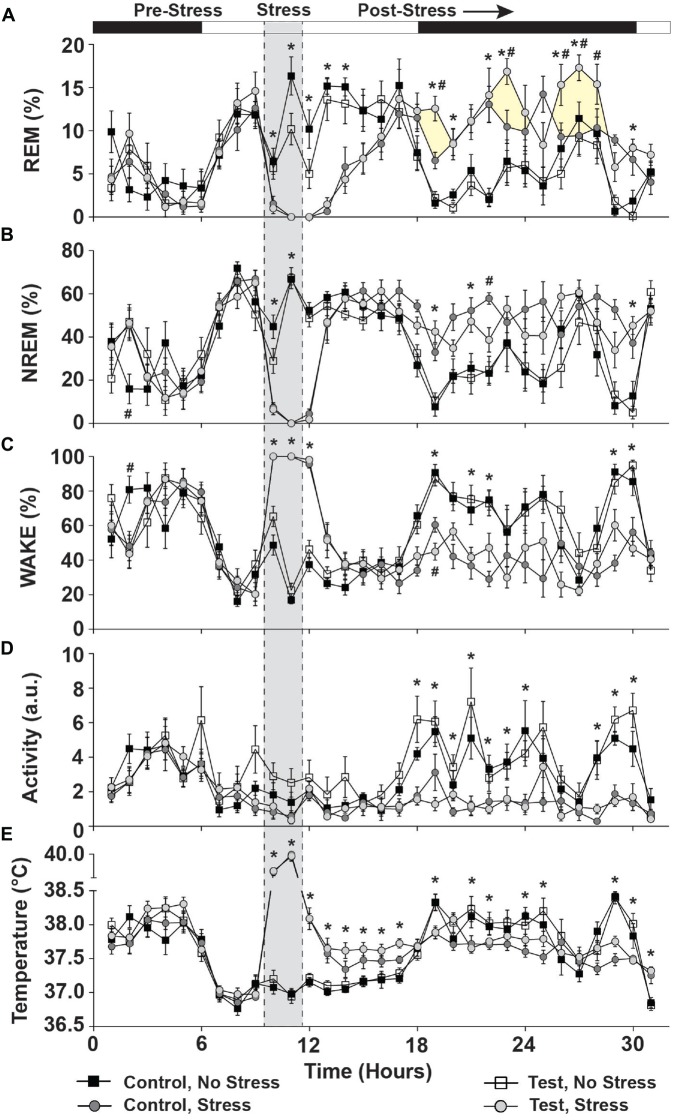
**Day of stress.** Data depicting that stress significantly disrupted sleep and physiology and that test diet enhanced REM sleep rebound following stress exposure. **(A)** REM sleep was abolished during stress and increased during the ensuing dark cycle. Rats consuming the test diet displayed enhanced REM rebound (yellow shading for clarity) when compared to stress rats eating the control diet. **(B)** NREM sleep was also abolished during stress and was increased compared to non-stressed rats in the ensuing dark cycle. **(C)** Wake was at a maximum during stress exposure and stressed rats spent less time awake during the ensuing dark cycle (due to increased sleep). **(D)** Rats exposed to stress also were less active during the nocturnal cycle following stress. **(E)** Stress induced a hyperthermic response in rats, which persisted for several hours following stress during the remaining light cycle, but was reversed to below the non-stressed controls upon the dark cycle onset (likely due to increased sleep in the stressed rats). Abbreviations are as follows: a.u., arbitrary units; NREM, Non-rapid eye movement; REM, Rapid eye movement. Note: shaded area of graph is for clarification and does not represent “actual” time stressed (see “Materials and Methods” Section) (*p < 0.05 vs. no stress; ^#^*p* < 0.05 vs. control diet).

There were no significant differences due to diet or between stress groups in the 10 h prior to stress exposure in REM sleep (Figure 5A; see pre-stress) NREM sleep (Figure 5B; see pre-stress) or Wake (Figure 5C; see pre-stress; see also Supplementary Figure S1). There was, however, a small time by diet interaction in NREM sleep (*F*_(1,25)_ = 2.433; *p* = 0.015; see # pre-stress Figure 5B for results of *post hoc* analysis) and in Wake (*F*_(1,25)_ = 2.338; *p* = 0.020; see # pre-stress Figure 5C for results of *post hoc* analysis) in the 10 h prior to stress exposure.

During exposure to stress REM sleep was abolished (*F*_(1,25)_ = 118.885; *p* < 0.0001; Figure 5A; see stress) and NREM sleep was eliminated (*F*_(1,25)_ = 661.855; *p* < 0.0001; see Figure 5B stress). During stress, wake was at a maximum (*F*_(1,25)_ = 948.620; *p* < 0.0001; see Figure 5C stress).

In the 20 h following stress (Post-stress, Figure 5), there was no main effect of diet on REM sleep, but a significant main effect of stress exposure (*F*_(1,25)_ = 13.581; *p* = 0.001; Figure 5A; see post-stress) and a significant diet by stress interaction (*F*_(1,25)_ = 4.942; *p* = 0.035; see post-stress Figure 5A for results of *post hoc* analysis), which was primarily due to beneficial dark cycle REM rebound sleep. NREM sleep was also altered by stress exposure (*F*_(1,25)_ = 62.196; *p* < 0.0001; Figure 5B; see post-stress) and there was a small, but significant, 3-way interaction between time, diet and stress, which is driven primarily by the effect of stress (*F*_(1,25)_ = 1.633; *p* = 0.044; Figure 5B for results of *post hoc* analysis; see post-stress). Wake was also altered following stress (*F*_(1,25)_ = 70.415; *p* < 0.0001; Figure 5C; see post-stress) and there was a trend towards a 3-way interaction between time, diet and stress (*F*_(1,25)_ = 1.599; *p* = 0.052; Figure 5C for results of *post hoc* analysis; see post-stress).

### Stress, but Not Diet, Altered Locomotor Activity and Body Temperature Immediately Following Stress (PND 87)

There were no significant differences in LA due to diet or between stress groups in the 10 h prior to stress exposure (Figure 5D; see pre-stress) or body temperature (Figure 5E; see pre-stress).

During exposure to stress (Stress; Figure 5) LA was reduced (*F*_(1,25)_ = 7.466; *p* = 0.011; Figure 5D; see stress) but there was no effect of diet. Conversely, stress exposure significantly increased body temperature (*F*_(1,25)_ = 383.244; *p* < 0.001; Figure 5E; see stress) and this was further increased to a maximum in the second hour of stress exposure (*F*_(1,25)_ = 46.813; *p* < 0.0001; Figure 5E; see stress) with no effect of diet.

In the 20 h following stress (Post-stress, Figure 5), LA was the same regardless of diet, but prior stress exposure led to a suppression of nocturnal LA (*F*_(1,25)_ = 36.254; *p* < 0.0001; Figure 5D; see post-stress) for several hours (*F*_(1,25)_ = 5.051; *p* < 0.0001; Figure 5D; see post-stress). In the 20 h following stress (Post-stress, Figure 4), body temperature was higher in rats exposed to stress (*F*_(1,25)_ = 16.447; *p* < 0.0001; Figure 5E; see post-stress), but there was no diet by stress interaction.

### Test Diet did Not Shift High Abundance Phyla in the Gut Microbial Community Across Development

**Figure 6:**
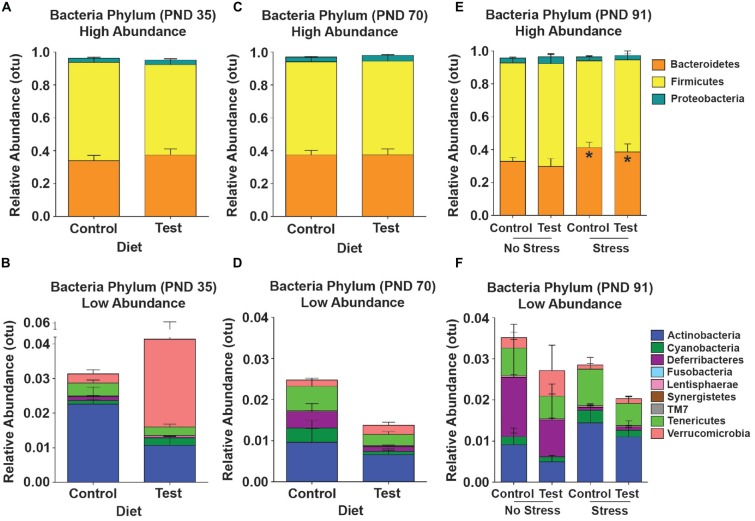
**16S rRNA data.** Taxonomic graphs depicting the relative abundance of phyla in the gut microbial communities at PND 35, PND 70 and PND 91. **(A)** The high abundance gut microbial community at PND 35 in rats consuming either the control or test diet. **(B)** The low abundance microbial communities depicted in the absence of bacteroidetes, firmicutes and proteobacteria. **(C)** The high abundance microbial community at PND 70 in rats consuming either the control or test diet. **(D)** The low abundance microbial communities depicted in the absence of bacteroidetes, firmicutes and proteobacteria. **(E)** The high abundance gut microbial community at PND 91 4 days after stress exposure in rats consuming either the control or test diet. There was a small, but significant, increase in the phylum bacteroidetes (orange bars). **(F)** The low abundance microbial communities depicted in the absence of bacteroidetes, firmicutes and proteobacteria 4 days after stress exposure. Abbreviations are as follows: OTUs, operational taxonomic units; PND, post natal days (*p < 0.05 vs. no stress groups).

Although there were minor fluctuations in the gut microbial community across development, there were no statistically significant shifts in high-abundance phyla across development due to consumption of the test diet (Figures 6A,C,E). There was, however, an increase in the lower-abundance phylum Deferribacteres (*F*_(1,25)_ = 9.843; *p* = 0.004; Figures 6B,D; purple) across time from PND 35 to PND 70 in both the control and test diet groups. At PND 91, 4 days after stress exposure, there was a significant increase in the high-abundance phylum Bacteroidetes (*F*_(1,23)_ = 4.917; *p* = 0.037; see Figure 6E, orange), but consumption of the test diet did not alter this stress-induced increase (Figure 6F).

### Test Diet Reduced the Impact of Stress in all Three Measures of Alpha Diversity

At PND 35 there were no significant differences in the three measures of alpha diversity: observed species (*F*_(1,25)_ = 0.009; *p* = 0.924; data not shown); Shannon entropy (*F*_(1,25)_ = 0.383; *p* = 0.542; data not shown) or PD whole tree analysis (*F*_(1,25)_ = 0.245; *p* = 0.625; data not shown) between either the control or test diets. Similarly, at PND 70 there were no significant differences in observed species (*F*_(1,25)_ = 0.146; *p* = 0.705; data not shown); Shannon entropy (*F*_(1,25)_ = 0.17; *p* = 0.897; data not shown) or PD whole tree analysis (*F*_(1,25)_ = 0.001; *p* = 0.926; data not shown) between the either the control or test diets. There was, however, a slight increase in Shannon entropy from PND 35 to PND 70 (*F*_(1,25)_ = 4.298; *p* = 0.048) regardless of control or test diet (6.33 ± 0.427 vs. 6.577 ± 0.496).

**Figure 7:**
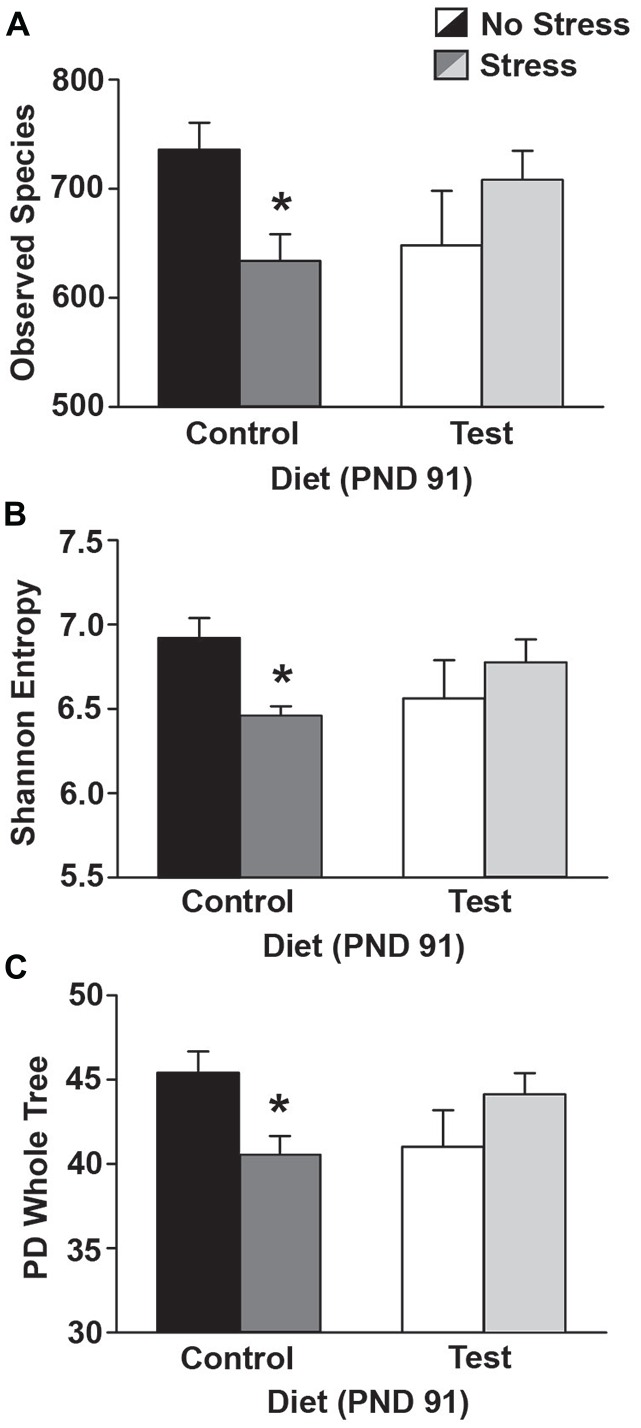
**Alpha diversity.** Data depicting three measures of alpha diversity. **(A)** Stress exposure reduced the number of observed species in rats on the control diet, but rats on the test diet were protected from this reduction in the number of observed species. **(B)** Stress exposure reduced Shannon Entropy in rats on the control diet, but this effect was attenuated in rats consuming the test diet. **(C)** Similarly, stress reduced PD whole tree analysis, but only in rats on the control diet. Abbreviations are as follows: PD, phylogenetic diversity; PND, post natal days (*p < 0.05 vs. control diet, no stress).

Four days following stress exposure at PND 91, there were significant interactions between diet and stress in the number of observed species (*F*_(1,23)_ = 7.067; *p* = 0.014; see Figure 7A for results of *post hoc* analysis), in Shannon entropy (*F*_(1,23)_ = 6.656; *p* = 0.016; see Figure 7B for results of *post hoc* analysis), and in PD whole tree analysis (*F*_(1,23)_ = 7.934; *p* = 0.009; see Figure 7C for results of *post hoc* analysis).

There were no shifts in Beta diversity at any age or under any conditioned examined (data not shown).

### Stepwise Multiple Regression Analyses Reveal Relationship between Early-Life Gut Bacteria and Sleep

Given that rats consuming the test diet had longer NREM episodes at PND 71, 72 (Figure 4C), we wanted to examine whether any gut microbiota from both PND 35 and PND 70 (see Figures 6A,B) could be used to predict the longer NREM episodes in rats consuming the test diet. Thus, we conducted a stepwise multiple regression analysis on all bacteria phyla and both time points PND 35, 70 to examine whether any individual or combination of gut bacteria phyla could, in part, help explain why the rats consuming test diet had longer NREM episode durations on PND 71, 72. A stepwise multiple regression (best-fit) analysis revealed a significant relationship with Deferribacteres at PND 35 and the NREM episode durations at PND 71, 72 (*F*_(1,27)_ = 4.561; *p* = 0.042; data not shown).

This suggests that although other variables (i.e., phyla, time points) were significantly correlated (i.e., Deferribacteres at PND 70) the stepwise multiple regression model was strengthened and thus more significantly correlated with Deferribacteres at PND 35. Given this significant relationship between Deferribacteres at PND 35 and NREM episode duration at PND 71, 72, we further examined whether this significant effect was specific to either the control or test diets.

**Figure 8:**
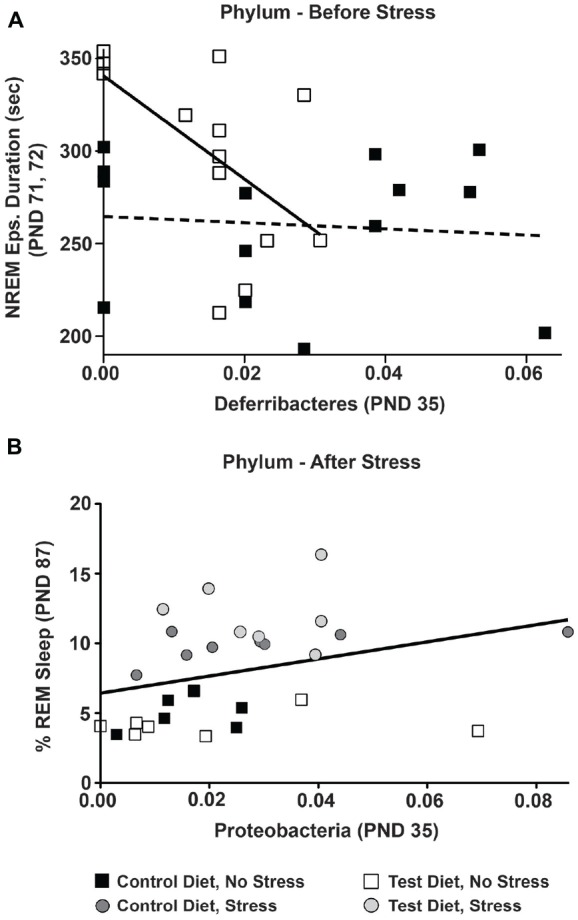
**Multiple regressions.** The relationship between early-life gut microbiota and sleep was examined by multiple regression analyses. **(A)** Prior to stress exposure, in the control diet group there was no significant relationship between Deferribacteres at (PND 35) and NREM episode duration at PND 71, 72 denoted by the dotted line. In the test diet group, however, there was a significant linear relationship between Deferribacteres and NREM episode duration such that rats consuming the test diet with lower levels of Deferribacteres (PND 35) had longer NREM episode durations denoted by the solid black line (*p* = 0.016). **(B)** Following stress exposure there was a small, but significant, linear relationship between Proteobacteria at PND 35 and REM recovery sleep at PND 87 denoted by the solid black line (*p* = 0.030). Abbreviations are as follows: PND, post natal days.

There was no significant relationship between Deferribacteres (PND 35) and NREM episode duration (PND 71, 72) in rats on the control diet (Figure 8A; dotted regression line), but there was a significant linear relationship between Deferribacteres (PND 35) and NREM episode duration (PND 71, 72) in rats consuming the test diet (*F*_(1,13)_ = 7.78; *p* = 0.016; Figure 8A; solid regression line). This significant relationship can be described by the equation: *y* = −2990.01*x* + 345.236.

There was also a small, but significant correlation between Proteobacteria at PND 35 and the REM sleep recovery 12 h after stress exposure (*F*_(1,27)_ = 5.239; *p* = 0.030; Figure 8B; solid line). Again, this suggests that although other variables (i.e., phyla, time points) were significantly correlated (i.e., Proteobacteria at PND 70) the stepwise multiple regression model was strengthened and thus more significantly correlated with Proteobacteria at PND 35. This significant relationship can be described by the equation: *y* = 23.763*x* + 4.488.

**Figure 9:**
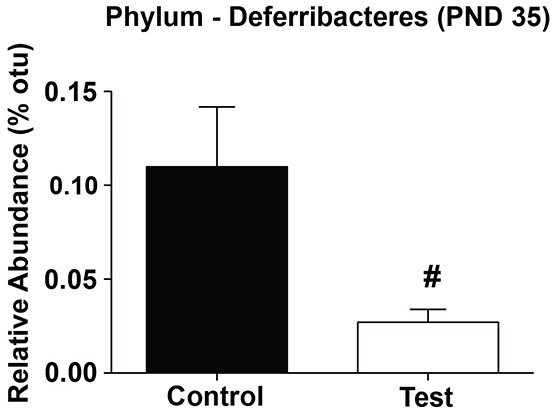
**Phylum Deferribacteres.** Rats consuming the test diet had significantly lower levels of Deferribacteres at PND 35 when compared to rats consuming the control diet. Abbreviations are as follows: OTUs, operational taxonomic units; PND, post natal days (^#^*p* < 0.05).

Finally, based on the linear relationship between Deferribacteres and NREM we examined whether there was a significant difference in the relative abundance levels of Deferribacteres on PND 35. Indeed, rats consuming the test diet had significantly lower levels of Deferribacteres at PND 35 when compared to rats consuming the control diet (*F*_(1,27)_ = 5.049; *p* = 0.033; Figure 9).

## Discussion

The results of the current study demonstrate that a (test) diet rich in prebiotics (GOS, PDX, Lf and MFGM) started in early life increases the growth of *Lactobacillus rhamnosus* and alleviates the stress-induced disruption of REM sleep, diurnal physiology and gut microbial alpha diversity. Rats on the test diet exhibited decreased impact of the stressor, including increased REM sleep rebound following stress, attenuated disruption of the diurnal rhythm of CBT, and prevention of dysbiosis in all three measures of alpha diversity. We also discovered that the test diet enhanced NREM sleep and this was related to changes in a specific phylum of bacteria (*Deferribacteres*) in early-life. Given that sufficient NREM sleep (Fogel et al., 2012; Novelli et al., 2013) and proper nutrition (Aboud and Yousafzai, 2015) can impact brain development and function and that sleep problems are common in early-life (Kempler et al., 2015), it is possible that a diet rich in prebiotics started in early-life could help improve sleep, support the gut microbiota and promote optimal brain/psychological health.

Test diet also modulated the impact of stress on sleep physiology. Rats eating test diet, compared to control diet, had increased time spent in REM sleep following stressor exposure or REM rebound. REM rebound is a well-characterized consequence of intense stressor exposure (Suchecki et al., 2012; Greenwood et al., 2014; Thompson et al., 2016) and is believed to be beneficial because it is associated with a reduced risk for developing post-traumatic stress disorder (Mellman et al., 2007). Consistent with the idea that prebiotics may be stress protective; several reports also suggest that individual prebiotics can reduce stress-evoked GI distress. For example, Hughes et al. (2011) reported that that 8 weeks of dietary GOS supplementation reduced GI stress in response to academic exams (Hughes et al., 2011), while a pre-clinical study found that dietary PDX administration can reduce intestinal colitis in response to trinitrobenzenesulfonic acid stress (Witaicenis et al., 2010). Our results, taken together with these studies, support the conclusion that dietary prebiotics can be stress protective.

Test diet not only affected sleep physiology, but also reduced the impact of stress on the diurnal rhythm of CBT. We have previously reported that stressor exposure flattens the diurnal amplitude of LA (Fleshner et al., 2013; Thompson et al., 2014), CBT (Thompson et al., 2013, 2014), and sleep (Greenwood et al., 2014; Thompson et al., 2016); and a prior history of repeated psychological stress can exacerbate these stress-induced diurnal disruptions (Thompson et al., 2012, 2014; Greenwood et al., 2014). In the current study, rats fed test diet had an attenuated impact of stressor exposure on CBT diurnal amplitude. Interestingly, people who ingested the prebiotic GOS had an attenuated waking cortisol response suggesting that prebiotics may also impact diurnal rhythms in humans (Schmidt et al., 2015). Thus these results suggest a diet rich in prebiotics can reduce the impact of stress on diurnal physiology.

Test diet prevented stress-induced gut microbial dysbiosis. Despite a lack of large individual phyla differences, the test diet prevented the stress-induced reduction in all three measures of microbial alpha diversity (i.e., number of species, Shannon entropy and phylogenic diversity whole tree). To our knowledge this is the first report to demonstrate that a test diet rich in prebiotics protects against the stress-induced reduction in alpha diversity. Alpha diversity is a measure of evenness and richness (Shannon, 1997; Faith and Baker, 2007) and low levels of alpha diversity are associated with increased vulnerability to mucosal inflammatory diseases later in life (Bisgaard et al., 2011; Voigt et al., 2014). Interestingly, a recent clinical study demonstrated that measures of Shannon entropy were positively correlated with sociability (Christian et al., 2015), suggesting that alpha diversity could also impact higher cognitive and emotional functions.

The stepwise multiple regression (best-fit) analysis revealed relationships between specific gut bacteria and sleep architecture. We found that reduced levels of early-life *Deferribacteres (PND 35)* in rats fed the test diet, is associated with a greater amount of NREM sleep later in life (PND 71,72). Importantly, this effect was transient as both groups had similar sleep/wake cycles by PND 85, 86 just before exposure to stress, suggesting a beneficial developmental effect. In addition, we discovered that higher levels of early-life *Proteobacteria (PND 35)* is associated with greater REM sleep recovery after stress. That both of our multiple regression analyses demonstrated early-life gut bacteria (PND 35) may influence the sleep/wake cycle later in life is consistent with previous literature suggesting that early-life gut bacteria may be more malleable and able to “set the stage” for health later in life (Mika et al., 2015a; Mika and Fleshner, 2016). The current data, although correlational, suggest specific bacteria may contribute to the sleep changes we observed.

Ingestion of prebiotics has been previously reported to increase several species of *Lactobaccilli*. Our results verify that ingestion of the test diet, compared to control diet, was effective at increasing *L. rhamnosus*. There are no previous studies, to our knowledge, testing the impact of *pre*biotics on sleep physiology; however, several studies have examined the impact of *pro*biotics. Diop et al. (2008), for example, tested 3 weeks of probiotic treatment using *Lactobacillus acidophilus Rosell-52 + Bifidobacterium longum Rosel-175* in male/female volunteers aged 18–60 who lived in France (Diop et al., 2008) and a second study administered *Lactobacillus reuteri* to infants for about 90 days (Cekola et al., 2015). Neither study found any demonstrable changes in sleep behavior; however, both studies lacked objective measures of sleep from polysomnogram recordings. Our current results, however, are consistent with a pre-clinical report demonstrating that administration of the probiotic *Lactobacillus brevis SBC8803* produced an increase in total time spent in NREM sleep during the light cycle in 6 week old mice (Miyazaki et al., 2014). Finally, a small clinical study from this same group recently reported that administration of heat killed *Lactobacillus brevis SBC8803* produced a small improvement in sleep in people with prior disrupted sleep (Nakakita et al., 2016). Thus, in light of our recent findings and previous work, it appears that administration of probiotics or modulation of the gut microbiota with a diet rich in prebiotics can impact NREM sleep. Clearly, more research is necessary to better understand how prebiotics affect the gut microbiota and how this relates to the sleep/wake cycle.

The bioactive milk fractions, Lf and MFGM, not only help promote growth of beneficial gut microbial species (Chatterton et al., 2013; Timby et al., 2015), but they may affect brain function directly. Clinical reports suggest that MFGM may enhance cognitive development and increase myelination when compared to controls (Tanaka et al., 2013). Lf is expressed in mature milk at levels of 1–2 g/L, and may be transported into the CSF by binding an Lf receptor on the choroid plexus (Talukder et al., 2003; Kamemori et al., 2008). Both Lf and MFGM have been reported to impact brain development and function (Mudd et al., 2016). Thus it may be possible that Lf and MFGM impact brain function directly, although this possibility needs further testing and remains unknown.

In conclusion, consumption of the test diet improved early-life NREM sleep and enhanced REM sleep rebound following stress. In addition, rats consuming the test diet had an attenuated stress-induced flattening of the diurnal rhythm of CBT and were protected from the stress-induced decrease in gut microbial alpha diversity. These data are the first to show that a diet rich in prebiotics can modulate the sleep/wake cycle both before and after stress and induce stress-protective effects in diurnal physiology and the gut microbiota. Our results, however, cannot address how increases in *Lactobacillus rhamnosus* or other changes in microbial community structure contribute to the observed effects of a diet rich in prebiotics and more work is necessary to further elucidate the potential mechanisms. Nonetheless, our work is the first to demonstrate that consumption of a prebiotic diet can provide stress protective effects on sleep/wake behavior.

## Author Contributions

RST and BNG designed, performed and analyzed the studies and wrote the manuscript. RR and AM conducted experiments, analyzed data and helped with the manuscript. RK conducted the 16S rRNA analysis and helped with the manuscript. MC and BMB helped with the design of the studies and offered comments on the manuscript. MF designed the studies and helped write the manuscript.

## Funding

This study was supported by grant funding from Mead Johnson Nutrition.

## Conflict of Interest Statement

Mead Johnson Nutrition reviewed and offered editorial suggestions for the manuscript, however, RST and Professor Fleshner had final control and approval of the manuscript content. MC and BMB are employees of Mead Johnson Nutrition. The other authors declare that the research was conducted in the absence of any commercial or financial relationships that could be construed as a potential conflict of interest.

## Supplementary Material

FIGURE S1 **Pre-Stress.** Supplemental data showing no significant main effects between groups in the later half of the light cycle into the first half of the dark cycle prior to stress exposure (i.e., PND 86). There were no significant differences in **(A)** %REM, **(B)** %NREM, **(C)** %Wake, **(D)** locomotor activity or **(E)** core body temperature.
