# Supplementary figures and images for: Dietary Prebiotics and Bioactive Milk Fractions Improve NREM Sleep, Enhance REM Sleep Rebound and Attenuate the Stress-Induced Decrease in Diurnal Temperature and Gut Microbial Alpha Diversity
Source: Front Behav Neurosci. 2017 Jan 10;10:240. doi: 10.3389/fnbeh.2016.00240 (PMC5223485; doi:10.3389/fnbeh.2016.00240)

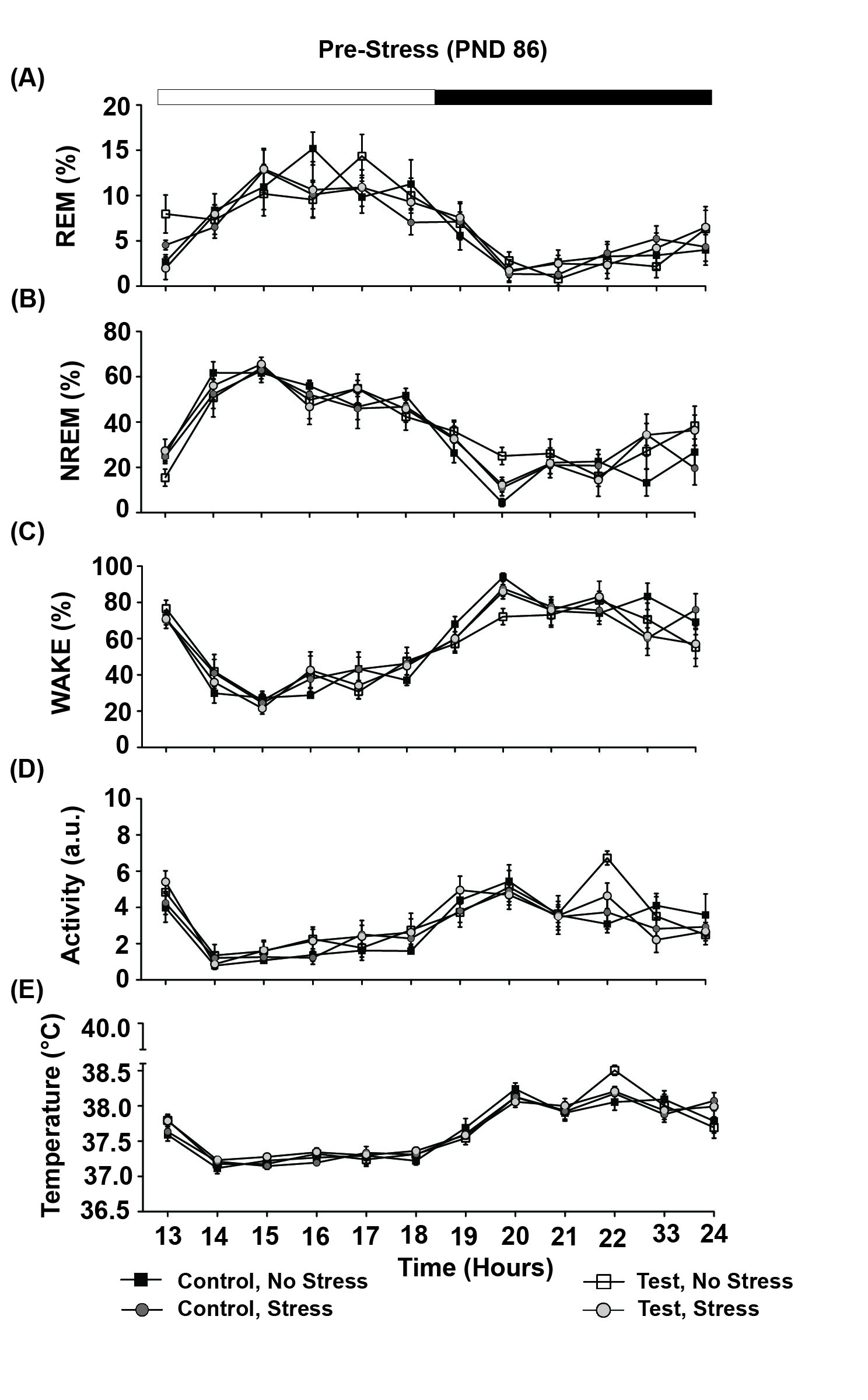

Supplement: FIGURE S1 — Pre-Stress. Supplemental data showing no significant main effects between groups in the later half of the light cycle into the first half of the dark cycle prior to stress exposure (i.e., PND 86). There were no significant differences in (A) %REM, (B) %NREM, (C) %Wake, (D) locomotor activity or (E) core body temperature. [file Image_1.jpeg]
